# Development of Orange Peel-Derived Alginate-Pectin Edible Coatings with Chitosan and Clove Essential Oil for Postharvest Preservation of Cherry Tomatoes

**DOI:** 10.3390/molecules31183347

**Published:** 2026-09-21

**Authors:** Athira Thekkath, Michelle Ji Yeon Yoo

**Affiliations:** School of Science, Auckland University of Technology, Auckland 1010, New Zealand

**Keywords:** edible coating, sodium alginate, pectin, chitosan, clove essential oil, orange peel valorisation, postharvest preservation, cherry tomato, antioxidant activity, DPPH

## Abstract

Postharvest losses of fresh produce remain a major global challenge, with fruits and vegetables estimated to suffer losses of 20–40% worldwide. This study developed sodium alginate-based edible coatings incorporating pectin and bioactive compounds derived from orange peel, chitosan, and clove essential oil for postharvest preservation of cherry tomatoes. Orange peel was utilised as a dual-purpose raw material for simultaneous extraction of pectin and bioactive compounds, supporting a sustainable dual valorisation strategy aligned with circular bioeconomy principles. The use of chitosan significantly reduced film moisture content, indicating denser polymer network formation with lower water retention. FTIR analysis indicated intermolecular interactions among alginate, pectin, chitosan, and clove oil components through hydrogen bonding and electrostatic interactions. Mechanical analysis demonstrated that formulation modifications had minimal effect on Young’s modulus but significantly influenced peak force values, suggesting alterations in structural integrity and fracture resistance of the films. Incorporation of clove essential oil in F3 substantially improved DPPH radical scavenging activity relative to F1 and F2, and showed the highest total phenolic content (TPC) on a wet-weight basis, with the best overall visual quality among coated formulations observed when stored at 4 °C for 15 days. The uncoated control developed visible mould growth during storage. The developed coating systems show potential as sustainable alternatives for enhancing postharvest quality and extending the storage stability of fresh cherry tomatoes, pending quantitative postharvest validation.

## 1. Introduction

Postharvest losses of fresh fruits and vegetables remain a major challenge in global food systems, with losses estimated at 20–40% of production worldwide [1]. Tomatoes are particularly susceptible to postharvest deterioration owing to their thin epidermal structure, high respiration rate, and elevated moisture content, which collectively accelerate moisture loss, microbial spoilage, and oxidative degradation, significantly reducing both the nutritional and economic value of fresh produce throughout the supply chain.

Cherry tomatoes (*Solanum lycopersicum* var. cerasiforme) are among the most commercially significant tomato varieties, valued for their distinctive flavour, high nutritional content—including lycopene, vitamin C, and polyphenolic compounds—and broad consumer appeal [2,3]. Their small size and high surface-area-to-volume ratio render them particularly vulnerable to postharvest deterioration relative to larger tomato varieties, with a reported shelf life of 7–14 days under refrigerated conditions. Moisture loss, enzymatic softening, and surface microbial colonisation are the primary drivers of quality decline, making cherry tomatoes a relevant and commercially important model system for evaluating edible coating performance.

Conventional preservation strategies, including synthetic fungicides, wax coatings, and petroleum-based packaging, have long been used to extend shelf life; however, growing concerns regarding chemical residues, non-biodegradable plastic waste, environmental pollution, and consumer demand for minimally processed, clean-label foods have driven the development of safer and more sustainable alternatives [4]. Edible coatings have emerged as promising postharvest technology because they form thin, semi-permeable barriers on food surfaces that reduce moisture loss, regulate gas exchange, and serve as carriers for bioactive compounds such as antioxidants and antimicrobials [5]. Unlike conventional packaging, edible coatings are biodegradable, environmentally compatible, and consumed with the product without generating additional packaging waste, aligning with Sustainable Development Goal 12.3 and national sustainability commitments [6].

The functional performance of edible coatings depends not only on the film-forming biopolymer selected but also on the bioactive compounds incorporated into the matrix [5]. Sodium alginate is widely used as a primary film-forming biopolymer because of its excellent film-forming ability, biocompatibility, and biodegradability [7]. Pectin is a complementary polysaccharide that contributes structural reinforcement and barrier properties when combined with alginate [8]. Pectin-based edible coatings have been reported to reduce moisture loss, respiration rate, and senescence in fresh produce, thereby contributing to improved postharvest quality and shelf-life extension [8]. Chitosan, a cationic polysaccharide, forms polyelectrolyte complexes with anionic alginate and pectin through electrostatic interactions, potentially improving film compactness and moisture barrier performance [9]. Clove essential oil, rich in the phenolic compound eugenol, provides strong antioxidant bioactivity and has been incorporated into edible coating systems to enhance functional preservation performance [10]. Clove essential oil was selected over other commonly studied essential oils, such as thyme, oregano, and cinnamon, because of its exceptionally high eugenol content, its established efficacy at low concentrations in food preservation applications, and its documented compatibility with polysaccharide-based film matrices [10].

Citrus processing industries generate substantial quantities of peel waste, accounting for approximately 40–50% of total fruit weight [11]. Orange peel represents a rich source of both pectin (approximately 20–30% of dry weight) and bioactive phenolic compounds including hesperidin, narirutin, and hydroxycinnamic acid derivatives [12]. The simultaneous recovery of both materials from a single agricultural by-product, rather than sourcing them separately, constitutes a dual valorisation strategy with clear sustainability advantages.

Despite extensive investigation of individual coating components, studies combining orange peel-derived pectin and bioactive extract within the same alginate–chitosan–essential oil system remain limited. Furthermore, most multi-component coating studies evaluate a final composite without systematically isolating the contribution of each ingredient. The stepwise formulation approach adopted in this study addresses this gap. The specific objectives of this study were to (i) extract pectin and bioactive compounds from orange peel and evaluate the recovered materials; (ii) develop three sodium alginate-based edible coating formulations using a stepwise strategy (F1: alginate–pectin base; F2: F1 with chitosan; F3: F2 with clove essential oil); (iii) characterise the physicochemical, mechanical, and antioxidant properties of the resulting films; and (iv) evaluate the postharvest preservation potential of the coatings through structured visual observation of cherry tomatoes stored at 4 °C for 15 days.

## 2. Results and Discussion

### 2.1. Visual Appearance of Edible Films

**Figure 1:**
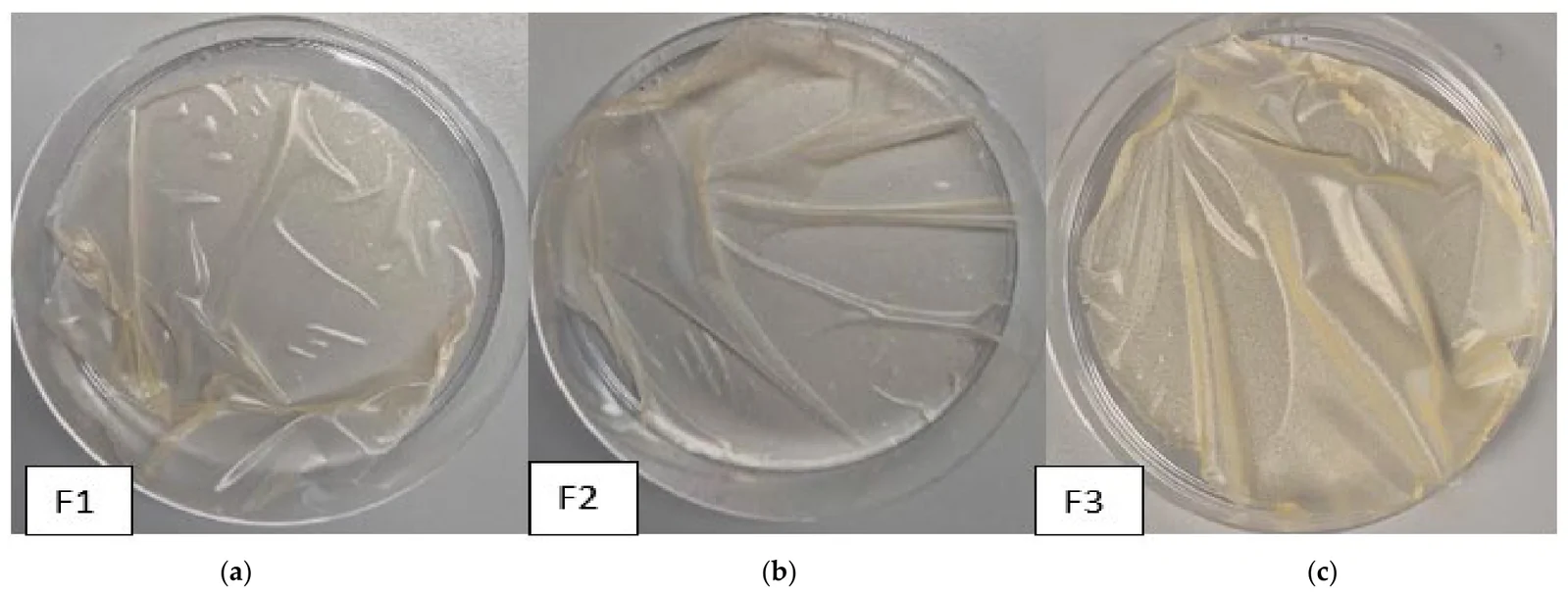
Representative photographs of edible films prepared from different coating formulations: (**a**) F1, (**b**) F2, and (**c**) F3.

Representative photographs of edible films prepared from different formulations are shown in Figure 1. All formulations successfully formed continuous films without visible cracking or phase separation. Differences in film appearance and transparency were observed among the formulations. Films containing chitosan and clove essential oil appeared slightly more compact and less transparent compared with the base formulation, which may be associated with increased intermolecular interactions within the polymer matrix.

### 2.2. pH

All formulations exhibited acidic pH values, with a significant and progressive decrease from F1 (4.07 ± 0.05) to F2 (3.78 ± 0.04) and F3 (3.61 ± 0.07) (ANOVA: F(2, 6) = 57.20, *p* = 0.000124; Table 1). The reduction in pH following chitosan incorporation in F2 is attributable to residual acidity from the citric acid used in chitosan dissolution, while the further decrease in F3 may reflect phenolic constituents of clove essential oil influencing matrix acidity. All formulations exhibited acidic pH values within a range commonly reported for polysaccharide-based edible coating systems. Acidic pH conditions are advantageous in edible coating systems as they may suppress microbial growth by limiting the environmental conditions favourable for spoilage organisms. Additionally, acidic conditions promote electrostatic interactions between the positively charged chitosan amino groups (−NH_3_^+^) and the negatively charged carboxylate groups (−COO^−^) of alginate and pectin, thereby contributing to polyelectrolyte complex formation [9,13].

### 2.3. Colour

The films exhibited high L* value, indicating light-colored films (Table 2). The mean L* values were 91.83 ± 0.02 for F1, 91.17 ± 0.57 for F2, and 91.74 ± 0.23 for F3. The approximate ΔE* values relative to F1 were 0.00 for F1, 1.57 for F2, and 2.04 for F3. Because these values were estimated from displayed treatment means rather than replicate-level measurements, they are presented descriptively and were not subjected to statistical analysis. No statistically significant differences were observed among the formulations for L*, a*, or b* values (p > 0.05). Film transparency was not independently quantified; therefore, high L* values should not be interpreted as direct evidence of transparency [14].

### 2.4. Moisture Content

Moisture content differed significantly among formulations (F(2, 6) = 101.9, *p* = 2.34 × 10^−5^; Table 3). F1 showed the highest moisture content (46.30 ± 1.64%), while F2 (28.09 ± 2.05%) and F3 (28.03 ± 1.71%) were significantly lower and did not differ from each other (*p* < 0.05). The substantial reduction in moisture content following chitosan incorporation in F2 is attributed to polyelectrolyte complex formation between chitosan amino groups and the carboxylate groups of alginate and pectin, which reduce available hydrophilic sites for water binding and restrict moisture mobility within the matrix, consistent with polyelectrolyte complex formation reported for similar alginate–chitosan systems [9]. The addition of clove essential oil in F3 did not produce a further significant reduction in moisture content, suggesting that the hydrophobic oil phase at the concentration used did not substantially contribute to additional water exclusion at this formulation level. Lower moisture content may contribute to improved structural stability and may be associated with altered moisture-barrier behaviour; however, direct water vapour permeability measurements are required to confirm barrier performance [15].

### 2.5. Mechanical Properties

Formulation composition significantly influenced peak force and tensile strength (F(2, 6) = 13.562, *p* = 0.0059) but not Young’s modulus (*p* = 0.1612; Table 4). F1 exhibited the highest peak force (2.03 ± 0.25 N) and tensile strength (2.90 ± 0.36 MPa), consistent with the cohesive hydrogen-bonded structure of the continuous alginate–pectin polymer matrix. The substantial reduction in peak force and tensile strength following chitosan incorporation in F2 suggests partial disruption of the alginate–pectin network, possibly attributable to competitive interference between chitosan and existing intermolecular bonds at the polymer concentrations used or to localised microstructural heterogeneity arising from rapid polyelectrolyte complexation during film formation. A partial recovery in peak force and tensile strength was observed in F3, which may reflect structural contributions of the emulsified oil phase to the polymer network; however, the mechanism warrants further investigation using microscopic techniques. The low Young’s modulus observed for F3 (0.023 ± 0.006 MPa) indicates greater film flexibility, which is advantageous for coating curved fruit surfaces without cracking.

### 2.6. FTIR Spectroscopy

**Figure 2:**
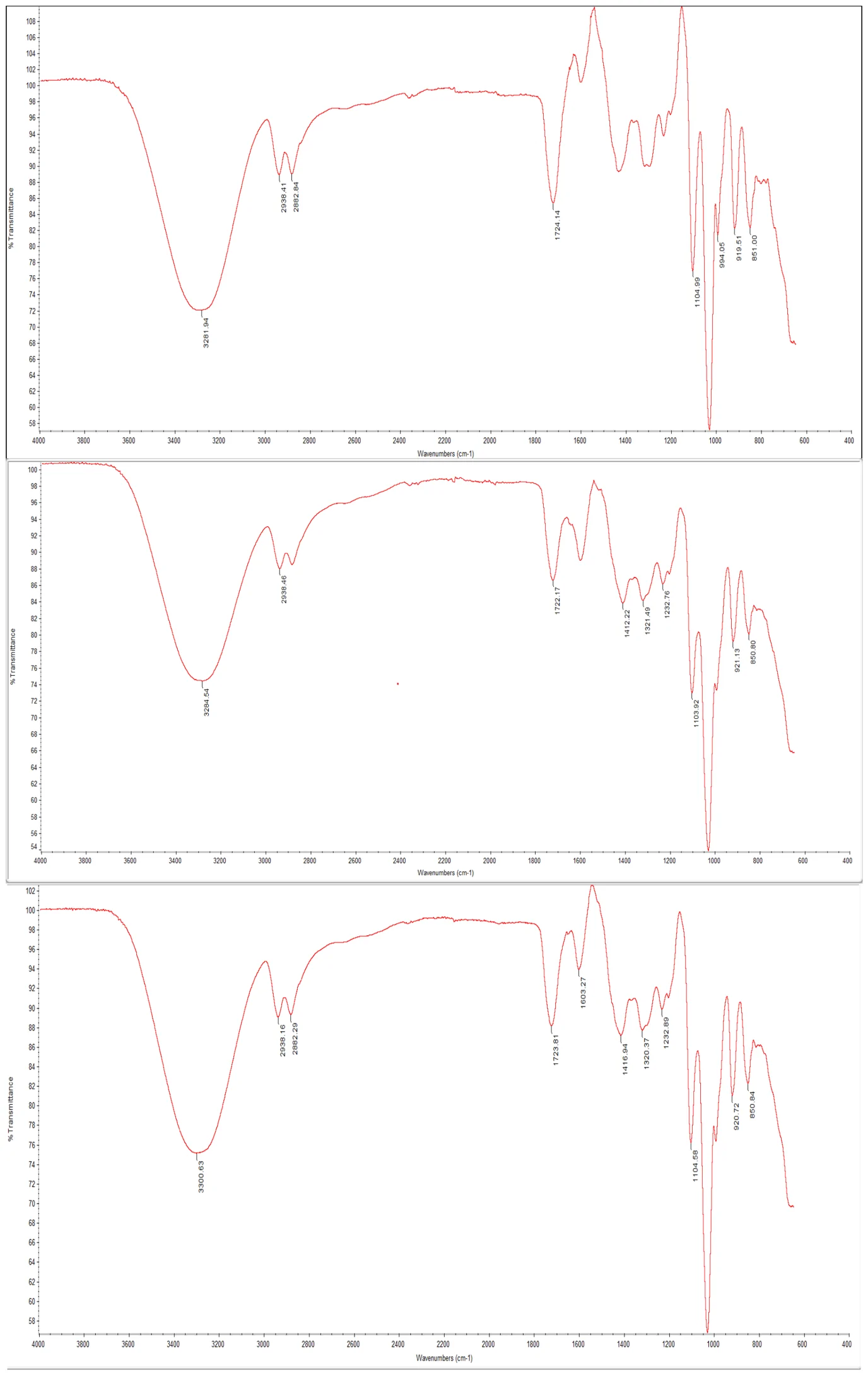
FTIR spectra of edible coating formulations F1, F2, and F3, recorded over 4000–400 cm^−1^.

FTIR spectra of all formulations (Figure 2) exhibited characteristic polysaccharide absorption bands: broad O–H stretching at ~3280–3300 cm^−1^, C–H stretching at ~2938 cm^−1^, C=O stretching at ~1722–1724 cm^−1^ (carboxyl and ester groups of alginate and pectin), and C–O–C glycosidic stretching at ~1031–1105 cm^−1^. In F2, modifications in the ~1600 cm^−1^ region indicated interactions between chitosan amino groups and alginate/pectin carboxylate groups, consistent with polyelectrolyte complex formation [9]. In F3, an additional absorption feature at ~1298 cm^−1^ was attributed to phenolic C–O stretching associated with eugenol in clove essential oil [10]. No new covalent bonds were detected; spectral changes among formulations are consistent with physical intermolecular interactions (hydrogen bonding and electrostatic associations) rather than chemical modification of polymer backbones [16].

### 2.7. DPPH Radical Scavenging Activity

All formulations demonstrated a dose-dependent increase in DPPH radical scavenging activity across the concentration range of 0.5–3.0 mg/mL (Table 5). One-way ANOVA confirmed highly significant differences among formulations at all four concentration points (*p* < 0.001), with F values ranging from 14,801 to 54,612, reflecting large and consistent between-group differences relative to within-group variability. Tukey’s HSD test confirmed that all three formulations differed significantly from each other at every concentration (*p* < 0.05).

**Figure 3:**
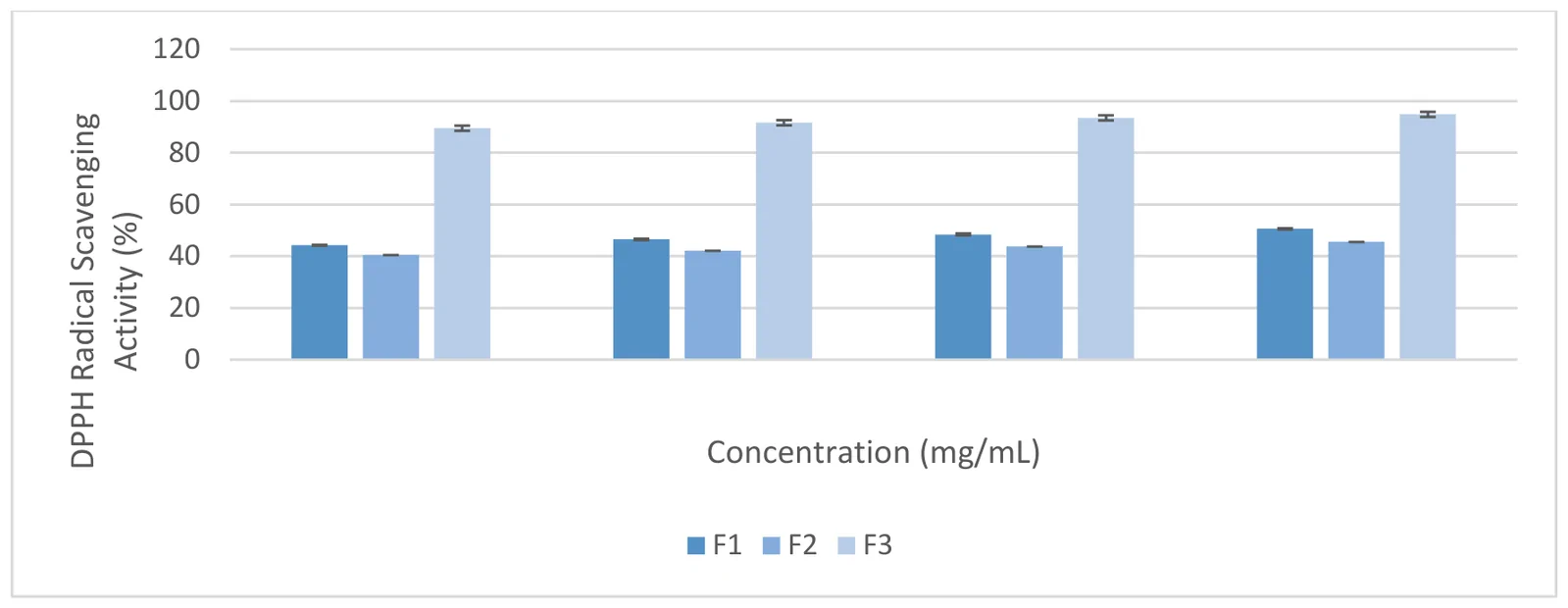
DPPH radical scavenging activity (%) of edible coating formulations F1, F2, and F3 as a function of concentration (0.5–3.0 mg/mL). Error bars represent standard deviation (*n* = 3). Different letters indicate significant differences among formulations at each concentration point (Tukey’s HSD, *p* < 0.05).

F3 exhibited the highest scavenging activity across all concentrations (89.46–94.82%), with an estimated IC_50_ below 0.5 mg/mL, indicating strong antioxidant capacity at low concentrations. This is primarily attributable to the phenolic compound eugenol in clove essential oil, which scavenges free radicals through hydrogen atom donation and electron transfer mechanisms [8]. These findings are consistent with the literature reporting high antioxidant activity for clove-enriched edible films [10]. F1 demonstrated moderate scavenging activity (44.24–50.58%; IC_50_ ≈ 2.73 mg/mL, estimated by linear interpolation), reflecting the contribution of citrus peel flavonoids in the orange peel extract [12]. F2 exhibited the lowest scavenging activity (40.46–45.52%; IC_50_ > 3.0 mg/mL) despite containing identical base ingredients to F1.The dose-response relationship among formulations is illustrated in Figure 3. This reduction may be attributed to chitosan–phenolic complexation, whereby electrostatic and hydrogen bonding interactions between chitosan amino groups and phenolic hydroxyl groups reduce the extractability and free radical reactivity of phenolic compounds during methanolic extraction [17]. This interpretation is supported by the corresponding reduction in TPC observed for F2.

### 2.8. Total Phenolic Content

TPC differed significantly among all three formulations (F(2, 6) = 6914, *p* = 8.16 × 10^−11^; Table 6), establishing the ranking F3 > F1 > F2, which was consistent with the DPPH antioxidant activity ranking. F3 exhibited the highest TPC (3.413 ± 0.012 mg GAE/g coating), attributable to phenolic compounds, particularly eugenol introduced by clove essential oil [10]. F1 showed intermediate TPC (2.874 ± 0.011 mg GAE/g coating), reflecting the contribution of orange peel extract phenolics. F2 exhibited the lowest TPC (2.329 ± 0.010 mg GAE/g coating) despite containing the same extract as F1, consistent with the chitosan–phenolic complexation mechanism described in Section 2.7 reducing phenolic extractability from the coating matrix [17]. The identical formulation ranking across both TPC and DPPH assays provides internal consistency to the antioxidant characterisation results, suggesting that phenolic compound content and availability were the primary determinants of antioxidant functionality across the three formulations. To account for differences in moisture content among formulations, TPC values were additionally expressed on a dry-weight basis: F1: 5.35 ± 0.02 mg GAE/g dry film; F2: 3.24 ± 0.01 mg GAE/g dry film; F3: 4.74 ± 0.02 mg GAE/g dry film. On a dry-weight basis, the ranking becomes F1 > F3 > F2, reflecting the higher intrinsic phenolic loading per unit of dry polymer matrix in F1 due to its substantially greater moisture content. The wet-weight basis ranking (F3 > F1 > F2) is considered more directly relevant to coating performance, as films are applied and stored with their actual moisture content; however, the dry-weight values confirm that all formulations contain meaningful phenolic content relative to their dry polymer matrix.

### 2.9. Storage Stability and Visual Quality Evaluation

**Figure 4:**
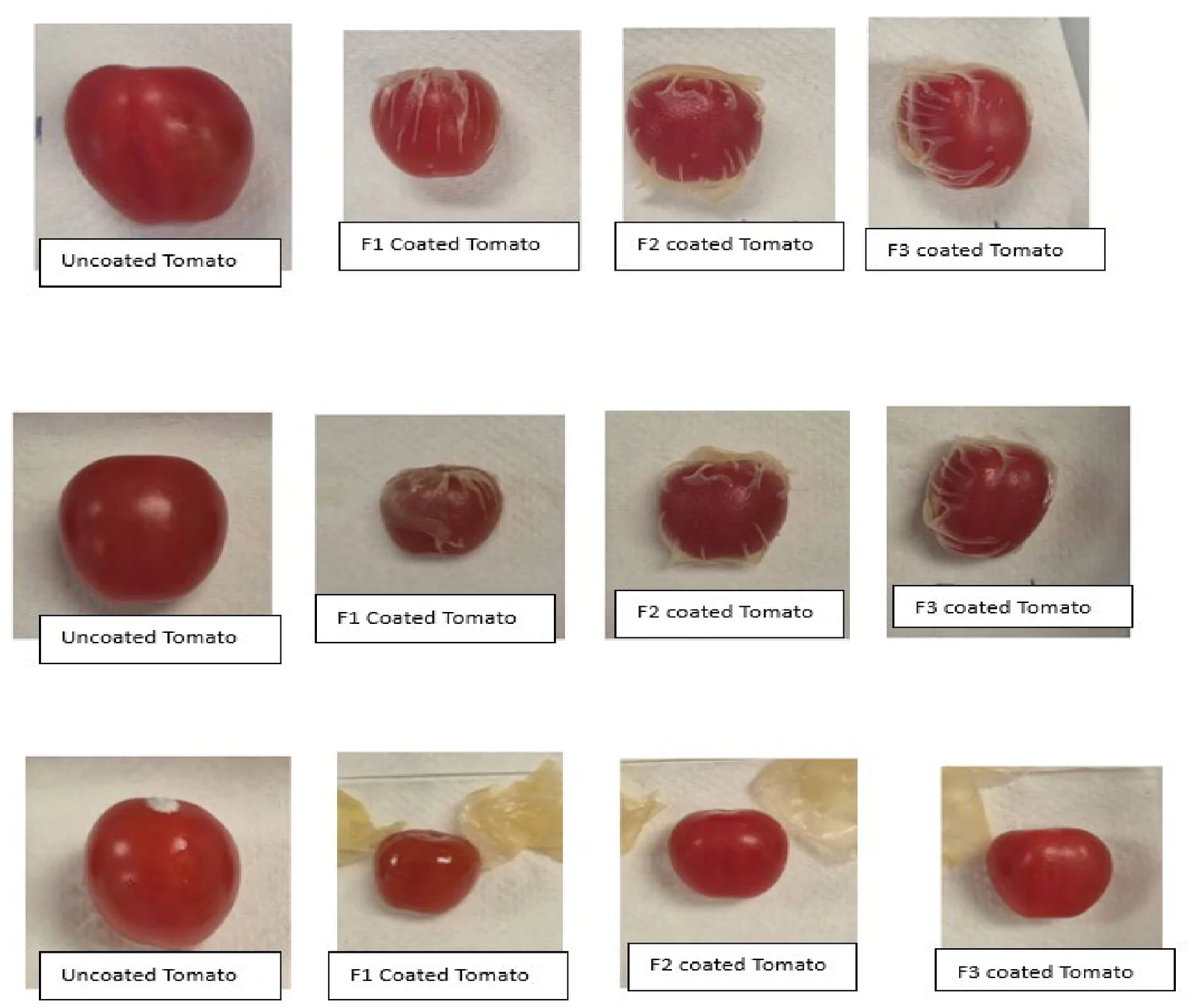
Visual appearance of uncoated control and coated cherry tomatoes (F1, F2, F3) during refrigerated storage at 4 °C on Day 1, Day 5, and Day 15 (*n* = 3 per treatment).

Visual quality assessment of cherry tomatoes stored at 4 °C for 15 days revealed differences in surface quality and spoilage progression among the treatment groups (Figure 4). Uncoated control tomatoes exhibited visible fungal growth and surface deterioration by Day 15, indicating spoilage development under refrigerated storage conditions. In contrast, tomatoes coated with F1, F2, and F3 remained free of visible mould throughout the observation period, suggesting that the physical barrier formed by the coating matrix may have contributed to limiting surface spoilage development. However, as no direct microbiological analysis was conducted, the potential role of antimicrobial compounds from chitosan or clove essential oil in this outcome cannot be confirmed from this study.

Among coated samples, F1-coated tomatoes exhibited noticeable softening during later storage stages, which may be associated with the comparatively higher moisture content (46.30%) and lower structural compactness of the F1 matrix. Tomatoes coated with F2 and F3 maintained better surface integrity and overall visual quality throughout the storage period, consistent with the denser polymer network formed following chitosan incorporation. Among all coated treatments, F3-coated tomatoes appeared to maintain the most favourable surface appearance among coated treatment groups based on visual assessment, with better retention of surface colour uniformity, turgor, and structural integrity through Day 15—an observation consistent with their superior antioxidant activity, lower Young’s modulus, and equivalent moisture reduction to F2 observed during physicochemical characterisation.

Gradual colour changes associated with normal ripening progression were observed across all samples during the storage period; however, coated tomatoes generally retained more acceptable visual quality relative to the uncoated control. These visual observations suggest potential coating effectiveness under the conditions evaluated, though quantitative confirmation is required. Future studies should incorporate quantitative postharvest measurements, including weight loss, instrumental firmness, respiration rate, titratable acidity, total soluble solids, and microbial enumeration, to fully characterise the preservation efficacy of these coating systems under both refrigerated and ambient storage conditions.

## 3. Materials and Methods

### 3.1. Materials

Orange peels were collected from a local juice franchise (Auckland, New Zealand) as a food-processing by-product. Fresh cherry tomatoes were purchased from a local supermarket (Auckland, New Zealand). Sodium alginate, chitosan, glycerol, citric acid, ethanol, Folin–Ciocalteu reagent, gallic acid, 2,2-diphenyl-1-picrylhydrazyl (DPPH), sodium carbonate, and Tween 80 were obtained from Sigma-Aldrich (St. Louis, MO, USA). Clove essential oil was purchased from a local chemist (Auckland, New Zealand). All reagents were of analytical grade.

### 3.2. Preparation of Orange Peel Powder

Fresh orange peels (1367 g) were washed and cut into small pieces using a sterile knife without removing the pith. The prepared peel pieces were rapidly frozen using food-grade liquid nitrogen and subsequently freeze-dried for 48 h using a Christ Alpha 1–4 LSCbasic freeze dryer (Martin Christ GmbH, Osterode am Harz, Germany). During the freeze-drying run, the instrument display indicated a temperature of −54.7 °C and a vacuum pressure of 0.609 mbar. Because the displayed temperature was recorded from the instrument screen and was not independently identified as the shelf, condenser, or product temperature, it is reported here as the temperature indicated during the freeze-drying process. The freeze-dried material yielded 328 g. A portion of this material (85 g) was ground and passed through a 150-mesh sieve. The powder was stored in sealed borosilicate glass containers under frozen conditions, and 40 g was used for extraction.

### 3.3. Extraction of Pectin and Bioactive Compounds

Pectin was extracted using acid-assisted hydrolysis adapted from Yapo [18] with minor modifications. Orange peel powder (40 g) was mixed with 400 mL of 4% (*w*/*v*) citric acid solution (pH 2.0) and heated at 80 °C for 90 min under continuous stirring to facilitate pectin solubilisation and extraction of water-soluble bioactive compounds. The extract was centrifuged and filtered through Whatman No. 1 filter paper. A 100 mL aliquot of the filtrate was retained as the orange peel bioactive extract. The remaining filtrate was precipitated with 400 mL of 95% ethanol, and the collected pectin was washed, freeze-dried for 24 h at a shelf temperature of −4 °C, and stored under refrigeration. A total of 9.8 g of dried pectin was recovered, corresponding to a yield of 24.5%.

### 3.4. Preparation of Edible Coating Formulations

Three formulations were prepared using a stepwise strategy. The concentrations of each component were selected based on values reported in the literature for similar polysaccharide-based edible coating systems [7,9,10]. Chitosan was incorporated at a concentration known to promote polyelectrolyte complex formation with alginate and pectin without adversely affecting film integrity [9]. Clove essential oil was added at a level consistent with reported effective concentrations in food coating applications [8], and Tween 80 was included as an emulsifier to facilitate stable oil dispersion within the aqueous matrix. For all formulations, 0.30 g sodium alginate was dissolved in 66.7 mL distilled water at 60 °C, followed by addition of 5.3 mL orange peel extract, 1.4 mL glycerol, and 33.3 mL pectin solution (1.5 g pectin in 100 mL distilled water). Formulation 1 (F1) comprised this base mixture. Formulation 2 (F2) incorporated 5.0 mL chitosan solution (1 g chitosan and 1 g citric acid in 100 mL distilled water), homogenised at 10,000 rpm for 3 min. Formulation 3 (F3) was prepared as F2, with the further addition of 0.3 mL clove essential oil pre-homogenised with 0.2 mL Tween 80 at 8000 rpm for 2 min. The solution (15 mL) was degassed for 10 min in an open glass vessel using a SONOREX SUPER RK 529(BANDELIN electronic GmbH & Co. KG; Berlin, Germany). ultrasonic bath operated continuously at a fixed frequency of 35 kHz and a nominal ultrasonic power of 60 W (240 W peak). Degassing was conducted at room temperature, and the solution was used for film casting immediately afterwards. Formulation compositions are presented in Table 7.

### 3.5. Film Casting and Drying

For physicochemical characterisation, 20 mL of each coating solution was poured into sterile 90 mm glass Petri dishes and dried in a hot-air oven at 37 °C for 16 h. The drying temperature of 37 °C was selected to avoid thermal degradation of heat-sensitive bioactive compounds while ensuring complete film formation, consistent with conditions reported for similar alginate-based edible films [7,9]. Films were carefully peeled off using a spatula and stored in a desiccator at room temperature prior to analysis.

### 3.6. pH Measurement

The pH of each coating solution was measured in triplicate prior to film casting using a calibrated digital pH meter (Eutech Instruments pH 700, Thermo Fisher Scientific, Singapore), calibrated with pH 4.0, 7.0 and 10.0 buffer solutions.

### 3.7. Colour Analysis

Colour properties of the dried films were measured in triplicate using a Nix Toolkit (Nix Sensor Ltd., Hamilton, Ontario, Canada) colour sensor against a white background. The colour parameters were recorded in the CIE Lab* colour space, where L* represents lightness, a* represents the red–green coordinate, and b* represents the yellow–blue coordinate. Total colour difference (ΔE*) was calculated using the CIE 1976 Lab* relationship described in ISO/CIE 11664-4:2019 [19].

$$\Delta E_{ab}^{*}=\sqrt{{\left(\Delta L^{*}\right)}^{2}+{\left(\Delta a^{*}\right)}^{2}+{\left(\Delta b^{*}\right)}^{2}}.$$

Formulation 1 (F1) was used as the reference sample. Because replicate-level colour data were unavailable, ΔE* values were estimated from the displayed mean L*, a*, and b* values. The approximate ΔE* values were 0.00 for F1, 1.57 for F2 relative to F1, and 2.04 for F3 relative to F1. These values are presented descriptively and were not subjected to statistical analysis.

### 3.8. Moisture Content Determination

Moisture content was determined in triplicate using the oven-drying method at 105 °C until a constant weight was achieved. The samples were dried using a Memmert UF160 universal laboratory oven (Memmert GmbH + Co. KG, Schwabach, Germany), rated at 230 V and 3200 W, with a maximum operating temperature of 300 °C.

Moisture content (%) = [(Wi − Wf)/Wi] × 100,

where Wi is the initial weight and Wf is the final dry weight.

### 3.9. Mechanical Property Measurement

Mechanical properties were evaluated in triplicate (*n* = 3) using a TA.XT Plus Texture Analyser (Stable Micro Systems Ltd., Godalming, UK) in tensile mode. Film strips (1 cm × 4 cm, ~0.07 mm average thickness) were tested at 1.00 mm/s with a 1 g trigger force. Peak force (N), Young’s modulus (MPa), and tensile strength (MPa; calculated as peak force divided by cross-sectional area of width × thickness = 0.70 mm^2^) were recorded.

### 3.10. FTIR Spectral Measurement

FTIR analysis was performed using a Nicolet iS10 FTIR spectrometer (Thermo Scientific, Madison, WI, USA) over 4000–400 cm^−1^ at room temperature. Characteristic absorption bands were assigned to functional groups associated with each coating component, and spectral differences among formulations were evaluated for evidence of intermolecular interactions.

### 3.11. Determination of DPPH Radical Scavenging Activity

Antioxidant activity was evaluated using the DPPH radical scavenging assay following a modified method of Divya et al. [20]. Film extracts were prepared by dissolving 0.1 g of each film in 10 mL methanol (stock concentration: 10 mg/mL). Aliquots (0.5–3.0 mL) were mixed with 0.1 mM DPPH solution and incubated in the dark for 1 h at room temperature. Absorbance was measured at 517 nm. Scavenging activity (%) = [(A_0_ − A_1_)/A_0_] × 100, where A_0_ is control absorbance and A_1_ is sample absorbance. Three independently prepared film samples per formulation were analysed (*n* = 3). The half maximal inhibitory concentration (IC_50)_ was estimated by linear interpolation where achievable.

### 3.12. Determination of Total Phenolic Content (TPC)

TPC was determined using the Folin–Ciocalteu colorimetric method. Film extracts (0.1 g in 10 mL methanol) were reacted with Folin–Ciocalteu reagent and sodium carbonate, incubated for 60 min in the dark at room temperature, and the absorbance was measured at 765 nm. A gallic acid calibration curve (y = 0.048x + 0.0346; R^2^ = 0.9998) was used to express TPC as mg gallic acid equivalents per gram of coating (mg GAE/g). Three independent film extracts per formulation (*n* = 3) were prepared.

### 3.13. Storage Study

Twelve uniform cherry tomatoes (three per treatment group) were surface cleaned with distilled water and air-dried at room temperature. Dried edible films were manually cut and pressed onto tomato surfaces to achieve complete surface coverage without wetting agents or adhesives, utilising the natural flexibility of the films to conform to the curved fruit surface. It is acknowledged that this pre-cast film application method differs from conventional liquid dip-coating and may influence interfacial adhesion and barrier dynamics; findings are therefore interpreted as indicative of coating matrix preservation potential. All tomatoes were stored in airtight containers at 4 °C for 15 days. Relative humidity within the storage environment was not monitored during the storage period; as tomatoes were stored in sealed airtight containers at 4 °C, the internal atmosphere is expected to have approached high-humidity conditions; however, relative humidity was not measured and direct comparison with commercial refrigerated storage conditions is therefore limited. The use of airtight containers at 4 °C was intended to simulate common refrigerated postharvest handling conditions for fresh tomatoes. Visual quality assessments were conducted on Days 1, 5, and 15 for mould development, surface colour changes, skin texture, and tactile softness. Findings are reported as qualitative descriptive observations under conditions representative of commercial refrigerated storage.

### 3.14. Statistical Analysis

All experiments were performed in triplicate. Data are expressed as mean ± standard deviation. One-way ANOVA followed by Tukey’s honestly significant difference (HSD) post hoc test was used to determine significant differences among formulations. For DPPH radical scavenging activity, one-way ANOVA was performed separately at each concentration point to compare formulations. Statistical significance was defined at *p* < 0.05. All analyses were performed using RStudio software version 2025.09.0+375 (Posit Software, PBC, Boston, MA, USA) with R version 4.5.1 (R Foundation for Statistical Computing, Vienna, Austria). The R Studio website is https://posit.co/ (accessed on 26 July 2026).

## 4. Conclusions

This study developed and characterised three sodium alginate-based edible coating formulations incorporating orange peel-derived pectin and bioactive extract, chitosan, and clove essential oil, and evaluated their physicochemical properties and preliminary postharvest preservation potential for cherry tomatoes. Orange peel was utilised as a dual-purpose raw material for simultaneous recovery of structural pectin and antioxidant-rich bioactive extract, supporting a dual valorisation approach aligned with circular bioeconomy principles, although further process optimisation studies would be needed to assess scalability.

Chitosan incorporation was associated with a significant reduction in film moisture content, while FTIR spectral changes were consistent with possible physical interactions among the film components. F3 exhibited the highest antioxidant performance, including DPPH radical-scavenging activity and wet-film TPC, which may be related to the addition of clove essential oil. However, F3 also contained Tween 80, so the individual contribution of clove essential oil cannot be isolated. The lower antioxidant activity and TPC observed in F2 relative to F1 may reflect altered phenolic extractability or accessibility after chitosan incorporation, but the specific mechanism was not directly verified.

Visual storage observations indicated that all coated tomatoes resisted visible mould development throughout the 15-day refrigerated storage period. As discussed in Section 3.9, F3 demonstrated the most favourable visual preservation outcome overall. These observations are interpreted as preliminary evidence of coating effectiveness pending microbial enumeration. Future work should incorporate weight loss, instrumental firmness, respiration rate, titratable acidity, total soluble solids, microbial enumeration, water activity, water vapour and oxygen permeability, and conventional liquid dip-coating trials. SEM imaging, HPLC quantification, and TPC/DPPH analysis of stored films are also needed to investigate microstructure and active-compound retention, degradation, migration, and release. The dual valorisation of orange peel as a source of both structural biopolymer and functional bioactive compounds represents a promising basis for developing sustainable active edible coatings for fresh produce preservation.

## Figures and Tables

**Table 1:** pH values of edible coating formulations expressed as mean ± standard deviation (*n* = 3), with ANOVA results.

Formulation	pH (Mean ± SD)	df	F-Value	*p*-Value
F1	4.07 ± 0.05 ^a^	2, 6	57.20	0.000124
F2	3.78 ± 0.04 ^b^			
F3	3.61 ± 0.07 ^c^			

Different superscript letters indicate significant differences according to Tukey’s HSD test (*p* < 0.05).

**Table 2:** Colour parameters of the edible film formulations. L*, a*, and b* values are expressed as mean ± standard deviation (*n* = 3); ΔE* values are approximate descriptive estimates relative to F1.

Formulation	L*	a*	b*	ΔE Relative to F1
F1	91.83 ± 0.02	−1.84 ± 0.02	6.33 ± 0.36	0.00
F2	91.17 ± 0.57	−2.05 ± 0.20	7.74 ± 1.19	1.57
F3	91.74 ± 0.23	−2.28 ± 0.31	8.32 ± 1.55	2.04

Values of L*, a*, and b* are presented as mean ± standard deviation (*n* = 3). ΔE* values were estimated from the displayed mean L*, a*, and b* values using F1 as the reference. Because replicate-level colour data were unavailable, standard deviations were not calculated for ΔE*; the values are, therefore, approximate and descriptive. No significant differences were observed among formulations for L*, a*, or b* values (*p* > 0.05). ΔE* values were not subjected to statistical analysis.

**Table 3:** Moisture content (%) of edible coating formulations, expressed as mean ± standard deviation (*n* = 3), with ANOVA results.

Formulation	Moisture Content (%)	df	F-Value	*p*-Value
F1	46.30 ± 1.64 ^a^	2, 6	101.9	<0.0001
F2	28.09 ± 2.05 ^b^			
F3	28.03 ± 1.71 ^b^			

Different superscript letters indicate significant differences according to Tukey’s HSD test (*p* < 0.05).

**Table 4:** Mechanical properties of edible coating formulations expressed as mean ± standard deviation (*n* = 3), with ANOVA results.

Parameter	F1	F2	F3	F-Value	*p*-Value
Young’s Modulus (MPa)	0.041 ± 0.008 ^a^	0.055 ± 0.028 ^a^	0.023 ± 0.006 ^a^	2.514	0.1612 (ns)
Peak Force (N)	2.03 ± 0.25 ^a^	0.44 ± 0.22 ^b^	0.85 ± 0.41 ^b^	13.562	0.0059
Tensile Strength (MPa)	2.90 ± 0.36 ^a^	0.63 ± 0.31 ^b^	1.21 ± 0.58 ^b^	13.562	0.0059

Different superscript letters within the same row indicate significant differences (Tukey’s HSD; *p* < 0.05). ns = not significant.

**Table 5:** DPPH radical scavenging activity (%) of edible coating formulations at each concentration point, expressed as mean ± standard deviation (*n* = 3), with ANOVA results.

Concentration (mg/mL)	F1 (%)	F2 (%)	F3 (%)	F-Value (df 2, 6)	*p*-Value
0.5	44.24 ± 0.18 ^a^	40.46 ± 0.28 ^b^	89.46 ± 0.46 ^c^	20,563	<0.0001
1.0	46.50 ± 0.28 ^a^	42.11 ± 0.27 ^b^	91.59 ± 0.55 ^c^	14,801	<0.0001
2.0	48.45 ± 0.37 ^a^	43.75 ± 0.28 ^b^	93.48 ± 0.38 ^c^	19,027	<0.0001
3.0	50.58 ± 0.21 ^a^	45.52 ± 0.18 ^b^	94.82 ± 0.21 ^c^	54,612	<0.0001

Different superscript letters within the same row indicate significant differences (Tukey’s HSD; *p* < 0.05).

**Table 6:** Total phenolic content (TPC) of edible coating formulations, expressed as mean ± standard deviation (*n* = 3), with ANOVA results.

Formulation	Absorbance (765 nm, Mean ± SD)	TPC (mg GAE/g, Mean ± SD)	F-Value	*p*-Value
F1	1.414 ± 0.006	2.874 ± 0.011 ^a^	6914	<0.0001
F2	1.153 ± 0.005	2.329 ± 0.010 ^b^		
F3	1.673 ± 0.006	3.413 ± 0.012 ^c^		

Different superscript letters indicate significant differences (Tukey’s HSD, *p* < 0.05). Gallic acid calibration equation: y = 0.048x + 0.0346; R^2^ = 0.9998.

**Table 7:** Composition of edible coating formulations (F1, F2, and F3).

Component	F1	F2	F3
Distilled water (mL)	66.7	66.7	66.7
Sodium alginate (g)	0.30	0.30	0.30
Orange peel extract (mL)	5.3	5.3	5.3
Glycerol (mL)	1.4	1.4	1.4
Pectin solution (mL)	33.3	33.3	33.3
Chitosan solution (mL)	—	5.0	5.0
Clove essential oil (mL)	—	—	0.3
Tween 80 (mL)	—	—	0.2

## Data Availability

The data presented in this study are available from the corresponding author upon reasonable request. The data are not publicly available because they form part of an ongoing research project.

## Abbreviations

The following abbreviations are used in this manuscript:

Abbreviation	Full Term
DPPH	2,2-Diphenyl-1-picrylhydrazyl
FTIR	Fourier Transform Infrared Spectroscopy
GAE	Gallic Acid Equivalents
IC_50_	Half-Maximal Inhibitory Concentration
TPC	Total Phenolic Content
WVP	Water Vapour Permeability
